# 10 European adder bites in Western Denmark - a case-report on the management in the emergency department

**DOI:** 10.1186/1757-7241-20-S2-P1

**Published:** 2012-04-16

**Authors:** Jesper Weile, Dorte Due-Rasmussen, Ulf Grue Hørlyk

**Affiliations:** 1 Emergency Department, Regional Hospital Herning, Denmark

## Background

The European Adder (vipera berus) is the most widely distributed poisonous snake in Europe. Bites by European adder are uncommon and correct management can reduce morbidity and mortality.

## Methods

We retrospectively reviewed all 10 adult patients treated in a rural emergency department over the summer of 2011 to assess the degree of morbidity, the cogency of treatment in the emergency department, and demographic data.

## Results

We found management ranging from no treatment (n=6) to antihistamine (n=3), corticosteroids (n=3), antibiotics (n=1), NON-SAIDs (n=3), heparin (n=2) and opioids (n=1). We found the most aggressive intervention was administrated in the two cases of highest level of severity. In cases of nil (n=5) or mild (n=3) affection only two were treated and both where graded nil. Hospital stay ranged from 1.5 hrs. to 120 hrs. (mean 27.75 hrs). We found no correlation between severity of envenomation and length of stay. Ages of patients spread from 25 to 69 (mean of 42.8). Three of the patients were German tourists. All patients recovered completely.

## Conclusion

Due to the discontinuity in the management in the emergency department we found it necessary to revise and present standardized guidelines for dealing with adder bites in the emergency department and prehospital setting.

As the number of encounters with such patients is very low we found it timely to produce a simple guideline for future management. We found it beneficial to distinguish between pre and in hospital treatment. Furthermore we found it necessary to list recommendations of what not to do as there are many falls perceptions in the field of immediate first aid in snakebites. Treatment with prednisone is controversial. We advocate that future studies should establish evidence based certainty in this field.

The limited size of sample group precludes statistical speculations on demographics.

We strongly advise emergency departments to revise and carry up to date literature and guidelines concerning bites by European adder. Due to the low number of patients we advise emergency departments in areas who are predisposed to receiving patients with adder bites to implement brief brush up courses on the subject early spring time every year.

